# Mutation analysis of the *MDM4 *gene in German breast cancer patients

**DOI:** 10.1186/1471-2407-8-52

**Published:** 2008-02-15

**Authors:** Scarlett Reincke, Lina Govbakh, Bettina Wilhelm, Haiyan Jin, Natalia Bogdanova, Michael Bremer, Johann H Karstens, Thilo Dörk

**Affiliations:** 1 Department of Gynaecology and Obstetrics, Hannover Medical School, Carl-Neuberg Str. 1, 30625 Hannover, Germany; 2 Department of Radiation Oncology, Hannover Medical School, Carl-Neuberg Str. 1, 30625 Hannover, Germany

## Abstract

**Background:**

MDM4 is a negative regulator of p53 and cooperates with MDM2 in the cellular response to DNA damage. It is unknown, however, whether *MDM4 *gene alterations play some role in the inherited component of breast cancer susceptibility.

**Methods:**

We sequenced the whole *MDM4 *coding region and flanking untranslated regions in genomic DNA samples obtained from 40 German patients with familial breast cancer. Selected variants were subsequently screened by RFLP-based assays in an extended set of breast cancer cases and controls.

**Results:**

Our resequencing study uncovered two *MDM4 *coding variants in 4/40 patients. Three patients carried a silent substitution at codon 74 that was linked with another rare variant in the 5'UTR. No association of this allele with breast cancer was found in a subsequent screening of 133 patients with bilateral breast cancer and 136 controls. The fourth patient was heterozygous for the missense substitution D153G which is located in a less conserved region of the MDM4 protein but may affect a predicted phosphorylation site. The D153G substitution only partially segregated with breast cancer in the family and was not identified on additional 680 chromosomes screened.

**Conclusion:**

This study did not reveal clearly pathogenic mutations although it uncovered two new unclassified variants at a low frequency. We conclude that there is no evidence for a major role of *MDM4 *coding variants in the inherited susceptibility towards breast cancer in German patients.

## Background

As part of a genome surveillance network, the tumour suppressor protein p53 becomes stabilized after DNA damage and modulates intracellular responses such as cell cycle arrest, DNA repair, senescence or apoptosis [1-3]. Multiple mechanisms regulate the activity of p53 at the posttranscriptional level [4,5]. One important antagonist, MDM2, is essential for ubiquitylation and subsequent degradation of p53 to maintain it at low levels in unstressed cells [6]. An MDM2-related protein, MDM4, has more recently emerged as another p53-interacting protein with a central role in the DNA damage response [7-9].

MDM4, also known as MDMX, is a 490 amino acid protein that is structurally related to MDM2 and binds to both, p53 and MDM2 [8]. MDM4 is regarded a negative regulator of p53 and cooperates with MDM2 to antagonize p53 [8-10]. In response to DNA double strand breaks, MDM4 becomes phosphorylated by the ATM and Chk2 kinases in an ATM-dependent manner which leads to a switch from the degradation of p53 to the degradation of MDM4 and consecutive stabilization of p53 [9,11-14].

Disruption of the p53 pathway is a key event in mammary tumorigenesis, and MDM4 is overexpressed in some 19% of breast carcinomas [15]. It is unknown, however, whether the *MDM4 *gene plays some role in the inherited component of breast cancer susceptibility. In the present study, we investigated the mutational spectrum of the whole *MDM4 *coding sequence in a group of German patients with familial breast cancer.

## Methods

### Patients

Our study population consists of a hospital-based series of 1012 unselected breast cancer patients who were treated at the Department of Radiation Oncology at Hannover Medical School from 1996–2001. Median age at onset of breast cancer was 57 years in this patient group, and 157 patients (15.8%) reported at least one first-degree relative with breast cancer. The patient series had been used previously to determine the frequency of selected mutations in the *BRCA1, ATM *and *CHEK2 *genes [16-20] as well as some more common polymorphisms in candidate genes tested by the Breast Cancer Association Consortium [21-23]. Forty patients were selected for the *MDM4 *resequencing study on the basis of (i) a family history of two or more first-degree relatives with breast cancer, or (ii) an age at onset of breast cancer below 50 years plus a family history of one first degree relative with breast cancer. Patients who were known carriers of a *BRCA1 *or *BRCA2 *mutation were not included into this study. The median age at onset for the 40 patients selected for the sequencing study was 48 years. Population controls were randomly taken from a consecutive series of anonymous female German blood donors recruited in 2005 at the same hospital. Written informed consent was obtained from each patient, and the study was approved by the Ethics commission at Hannover Medical School.

### Mutation analyses

Genomic DNA was isolated from peripheral EDTA blood samples using standard phenol-chloroform extraction. All exons of the *MDM4 *gene were amplified by polymerase chain reaction using primer pairs with sequences flanking the respective exons (Table 1, Genbank NT_004487.18). 35 cycles of PCR were carried out using HotStart Taq DNA Polymerase (Qiagen) with 1 min annealing at the primer specific temperature (Table 1), 1 min extension at 72°C and 1 min denaturation at 94°C. Sequencing reactions were performed using BigDye v1.1 chemistry, and sequences were evaluated on a Genetic Analyzer 3100 Avant (Applied Biosystems). Sequencing primers were the same as the PCR primers (Table 1). The genomic region covering exon 1 of the *MDM4 *gene was additionally amplified in 133 breast cancer and 136 control samples to allow for a subsequent restriction-enzyme based screening of the c.-103 T/C variant using *Mbo*I (New England BioLabs). Reaction products were separated on a 2% agarose gel and were evaluated by staining with GelRed. In the presence of the c.-103T allele, PCR product was cleaved to fragments of 129 bp and 282 bp, whereas product from the C allele remained at 411 bp length. The genomic region covering exon 7 of the *MDM4 *gene was additionally amplified in 140 breast cancer and 200 control samples to allow for a subsequent restriction-enzyme based screening of the D153G mutation using *Bbs*I (New England BioLabs). Reaction products were separated on a 3% agarose gel and were evaluated by staining with GelRed. In the presence of the D153G mutation, the 334 bp wildtype product was cleaved to fragments of 110 and 224 bp, whereas the mutant product remained uncut. Positive and negative controls were included into each assay and samples that remained uncut were subjected to direct sequencing to avoid false positives.

**Table 1:** Primers and PCR conditions used for *MDM4 *screening. Summary of primers, annealing temperatures and PCR product sizes for the eleven exons of the *MDM4 *gene. Forward and reverse primer sequences are listed. Exons 2 – 11 are coding exons. Exon 11 was amplified in two overlapping parts in order to make it more accessible to sequencing analysis.

**Exon**	**Primer**	**Annealing (°C)**	**Product size (bp)**
**1**	5'-TCTGATCTCCTAATACACGTCTG-3' 5'-GCCTGCTCCTCACTCTCCAG-3'	62	416
**2**	5'-CTGGTTGCCTTTGTGTGAATG-3' 5'-CTGGGATTACAGTCATGAGAC-3'	62	296
**3**	5'-AGAGGTTCTCTTGTTCCATAG-3' 5'-GATCTGAGATCGCACCACTG-3'	62	253
**4**	5'-CAGATCAGTTCATTTCTGTGCTG-3' 5'-CATATTCTCAGTGCCTCATAGG-3'	62	520
**5**	5'-GATTCTGCCTTTGTATGCCTTAC-3' 5'-CTCAAAGCTGTGATACAGACTG-3'	62	234
**6**	5'-CAGCCAACATGGAGAAGTAC-3' 5'-CAGAGAAGGTTCACTCTGTC-3'	62	356
**7**	5'-GGTGAGCCAGAATGGAACTG-3' 5'-ACAATGGTAGTAACTAGGCTG-3'	62	334
**8**	5'-AGCTCTGCCACTAAGACAG-3' 5'-CAAGAGTAACCAAGAATGTTACC-3'	62	389
**9**	5'-GATGTAGTGTGACGACATTGAG-3' 5'-CAACAGATGATACTATGAGCAC-3'	62	311
**10**	5'-CTACTACTGAAATGCCAACTAGAAG-3' 5'-GGATTACATCATCTGAAGATGG-3'	62	329
**11 (a)**	5'-GCTTAGTGAGAGGATGTGAATG-3' 5'-CTGTCGTTAGACCTAAAGATGC-3'	59	431
**11 (b)**	5'-CTGTCGTTAGACCTAAAGATGC-3' 5'-CCTAAGAACATTCTCTGACAG-3'	59	475

## Results

We established conditions to amplify the eleven exons of the *MDM4 *gene (Table 1). The whole coding region and flanking sequences were then analysed by direct sequencing in genomic DNA samples from 40 German breast cancer patients with a family history of disease. Eight distinct sequence alterations were identified (Table 2). Six of these were known polymorphisms listed in the NCBI SNP database with four of them located in flanking intron sequences, one in the 5'-UTR and one in the 3'-UTR. Maximum likelihood considerations indicated that the three SNPs in the introns 8, 9 and 10 were in absolute linkage disequilibrium, and that three common haplotypes could be defined on the basis of either the rare allele of rs4252697 in intron 5 (~16%), or the rare alleles of rs4252717-rs2290855-rs2290854 in the intron 8-9-10 block (~26%), or the common alleles at these four loci (~58%). Two less common alterations were identified in the coding region, the silent V74V substitution in 3/40 patients and the missense substitution D153G in a single case (Table 2).

**Table 2:** Genetic alterations of the *MDM4 *gene in 40 German patients with familial breast cancer. Survey of genetic alterations of the *MDM4 *gene identified in 40 patients with familial breast cancer. Mutations were designated according to the improved mutation nomenclature recommended by the Human Genome Variation Society [50]. Het, heterozygous; hom, homozygous. Variants c.-103T>C and c.222A>T were identified in the same three individuals. The three SNPs c.672+28C>T, c.823-62T>C and c.903+20G>A were identified in the same 19 individuals, with 17 of them also carrying c.*+32A>C.

Location	Nucleotide change	Codon	No. of carriers (frequency)			NCBI database
			het	hom	total	

5'-UTR	c.-103T>C	none	3 (.08)	-	3 (.08)	rs4252668
Exon 4	c.222A>T	Val74Val	3 (.08)	-	3 (.08)	not listed
Intron 5	c.343+9C>T	none	12 (.30)	1 (.03)	13 (.33)	rs4252697
Exon 7	c.458A>G	Asp153Gly	1 (.03)	-	1 (.03)	not listed
Intron 8	c.672+28C>T	none	19 (.48)	-	19 (.48)	rs4252717
Intron 9	c.823-62T>C	none	19 (.48)	-	19 (.48)	rs2290855
Intron 10	c.903+20G>A	none	19 (.48)	-	19 (.48)	rs2290854
3'-UTR	c.*+32A>C	none	17 (.43)	-	17 (.43)	rs4245739

The V74V substitution is a synonymous transversion c.222A>T that changes a GUA codon to GUU. It is located within a weak potential alternative splice donor site, however maximum entropy calculations predicted that this site is not improved by the substitution [24]. A search for exonic splicing enhancer sequences predicted that the substitution creates an additional binding site for the serine/arginine-rich (SR) protein SC35 which may improve exon recognition [25]. However, preliminary analyses of *MDM4 *mRNA in lymphoid cells did not reveal evidence for alternative splicing of exon 4 (data not shown), and this exon skipping is not among previously reported alternative splicing events [26,27]. On the basis of the available information, we considered a functional contribution of the V74V substitution unlikely and did not screen further for this substitution. Of some interest, all three patients who were heterozygous for V74V also carried the variant c.-103C>T in the 5'-UTR (rs4252668), suggesting that these two SNPs might be in linkage disequilibrium. Because SNPs in the 5'-UTR can exert regulatory functions in some genes with implications for breast cancer risk, e.g. in *RAD51 *[28-31], we assessed the frequency of the rare allele of rs4252668 in an additional set of 133 patients with bilateral breast cancer and 136 population controls. The rare allele was detected in 4/133 cases and 7/136 controls suggesting that it does not confer a significant increase in breast cancer risk (OR 0.6, 95% CI 0.2–2.0, p = 0.38).

**Figure 1:**
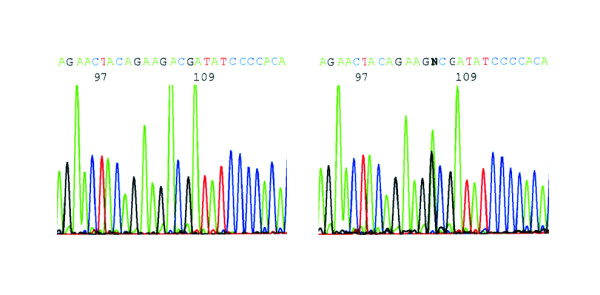
**Identification of the D153G mutation in exon 7 of the *MDM4 *gene**. Identification of the D153G mutation in exon 7 of the *MDM4 *gene by direct sequencing. Left: control with wildtype sequence, right: heterozygosity for c.458A>G (D153G).

The second nucleotide change in the coding region gives rise to a non-conservative amino acid substitution D153G (Figure 1) and was identified in only one patient with familial breast cancer. This patient had been diagnosed with unilateral breast cancer by the age of 24 years. Her mother had unilateral breast cancer by the age of 41 years and basal cell carcinoma by the age of 49 years, and the sister of the mother was diagnosed with unilateral breast cancer by the age of 44 years. We confirmed the D153G mutation in separate amplification reactions and also in the patient's mother but not in the maternal aunt, indicating an incomplete segregation pattern. The D153G substitution is embedded within a region of unknown structure and with no apparent homology to MDM2. A screen for evolutionary conservation with SIFT v.2.0 predicted this substitution to be tolerated [32]. A search for exonic splicing enhancers revealed that the D153G substitution is predicted to destroy a binding site for the SF2/ASF splicing factor [25]. However, only one of two overlapping binding sites is affected by the substitution suggesting that there might be no gross change in exon recognition. A PROSITE search for protein binding and phosphorylation sites revealed one possible motif, 150-TTED-153, which is a predicted casein kinase II (CK2) phosphorylation site [33]. We screened another 140 cases with bilateral breast cancer and 200 random female control individuals for the presence of the D153G mutation using a restriction enzyme-based assay. No further carrier was detected indicating that this missense substitution is very rare, at least in the German population.

## Discussion

Dysregulation of the p53 network is pivotal to mammary carcinogenesis, and germline alterations in the *TP53 *and *MDM2 *genes have been found to modulate the inherited risk of breast cancer [34-39]. The MDM4 protein associates with and regulates both proteins, p53 and Mdm2, in the DNA damage response pathway. It was therefore tempting to investigate whether activating germ-line mutations exist in the *MDM4 *coding sequence that could also contribute to breast cancer risk. We thus sequenced all exons and flanking non-coding sequences of *MDM4 *in 40 German patients with familial breast cancer. It is the first study, to our knowledge, that assesses the frequency distribution of *MDM4 *germ-line alterations in familial breast cancer.

Outside of the coding region, we confirmed common single nucleotide polymorphisms (SNPs) in the introns 5, 8, 9 and 10 as well as in the 5'- and 3' untranslated regions (UTR). Although we cannot assign any function to these SNPs, the SNPs in the untranslated regions may be useful to measure allelic imbalances at the mRNA level. We also noticed that there appeared to be a slight underrepresentation of rare homozygotes for the coupled intronic variants in IVS8, 9 and 10 as one may have expected 2–3 homozygotes among the sequenced cases. This is not a technical problem since we identified a homozygote among a few additional samples after limited sequencing of these portions of the gene. We think that it might be a spurious observation due to a relatively small number of fully sequenced samples but we cannot formally exclude the possibility that there is selection. The data provided here will enable subsequent studies that specifically address the distribution of the three main haplotypes and the corresponding genotypes in large case-control settings. The NCBI SNP database also lists SNPs in the coding region of *MDM4*, including two amino acid substitutions (I175T, T406I) and two synonymous changes (E303E, K468K) [40]. None of these are reported to surpass an allele frequency of 0.05, and so it is not surprising that none of them were found in our study. Instead, we identified two new single nucleotide variants in the coding region, V74V and D153G.

The V74V substitution is a synonymous transversion, but at least some synonymous changes can affect splicing, mRNA stability or translational efficiency [41]. *In silico *predictions for V74V did not provide strong support for a role in splicing, and single exon skipping was not observed as an alternative splicing event in *MDM4 *[26,27] Another possibility would be that the silent substitution acts at the translational level. Given that the GUA codon has been classified as a "translationally weak" codon that does not translate through wobble base-pairing, some stimulating effect of its replacement by GUU on the expression or on the folding properties of the MDM4 protein cannot finally be excluded [42-44]. However, both codons are commonly used in the *MDM4 *coding sequence and are represented seven and eight times, respectively. Because all three patients who were heterozygous for V74V also carried the variant c.-103C>T in the 5'-UTR (rs4252668), it remains possible that there is a regulatory function associated with this allele. However, the c.-103C>T variant is not predicted to target a transcription factor binding site when tested by the TFSearch program [45], and a screening of additional 269 individuals did not reveal differences between the carrier frequencies in cases and in controls. On the basis of all hitherto available information, we thus consider a functional or pathological contribution of the c.-103C>T – V74V allele unlikely.

The second nucleotide change in the coding region, c.458A>G, gives rise to a non-conservative amino acid substitution D153G and was identified in only one patient with familial breast cancer. There was only partial segregation of this substitution with breast cancer in the single family but such observations have also been made for other susceptibility alleles with low to moderate penetrance and do not exclude a possible contribution to breast cancer risk. The D153G substitution is located within a predicted casein kinase II (CK2) site. CK1 and CK2 are two protein kinases that participate in a wide variety of cellular processes, including DNA repair and cell cycle control, and phosphorylate Ser or Thr residues flanked by Asp, Glu, or phosphorylated Ser residues in the +3 or -3 position, respectively [46,47]. In some instances, phosphorylation by CK1 or CK2 may be required to license ATM substrate phosphorylation [48]. In MDM4, Ser289 has been reported as a phosphorylation site for CK1 while there is no report of MDM4 phosphorylation by CK2 at Thr150 or other sites [49]. Furthermore, there are multiple more CK2 sites predicted in the MDM4 sequence. Altogether, there is presently insufficient evidence to conclude that the D153G variant exerts any effect at the functional level. We did not find this substitution in an additional set of 140 cases with bilateral breast cancer and 200 random female control individuals indicating that it is very rare, at least in the German population. Therefore, its role for breast cancer, if any, might be very limited and probably hard to be defined in future association studies.

Altogether, this study did not support a major role for *MDM4 *coding variants in familial breast cancer risk. It remains possible that rare mutations exist which may have been missed due to the relatively small sample size of fully sequenced cases in our exploratory study. Furthermore, our families do not have extreme phenotypes and therefore may be more likely to harbour polygenic susceptibility alleles rather than highly penetrant mutations. It is also possible that more families with the D153G mutation may be detected in an extended case-control series. However, several thousands of samples would have to be screened to confirm, for instance, a 2–3 fold risk for a variant with a carrier frequency of less than 1%. The paucity of mutations in our study population seems to be consistent with the lack of evidence from breast cancer linkage studies for linkage to the *MDM4 *locus, and so highly penetrant founder mutations in the *MDM4 *gene might at best account for only a small proportion of German breast cancer patients.

## Conclusion

In summary, a resequencing study of the *MDM4 *gene in 40 German breast cancer patients selected for family history did not reveal clearly pathogenic mutations although it uncovered two new unclassified variants at a low frequency. We conclude that there is no evidence for a major role of *MDM4 *coding alterations in the inherited susceptibility towards breast cancer in German patients.

## Competing interests

The author(s) declare that they have no competing interests.

## Authors' contributions

SR established PCR and sequencing conditions to analyse the *MDM4 *coding region. SR, LG and BW performed the sequencing analyses. HJ and NB performed the case-control screening studies. MB and JHK provided blood samples and clinical records from the patients of the Department of Radiation Oncology. TD initiated and coordinated the study and drafted the manuscript. All authors took part in the critical discussion and proofreading of the manuscript.

## Pre-publication history

The pre-publication history for this paper can be accessed here:



## References

[B1] Lane DP (1992). p53, guardian of the genome. Nature.

[B2] Levine AJ (1997). p53, the cellular gatekeeper for growth and division. Cell.

[B3] Vousden KH, Lu X (2002). Live or let die: the cell's response to p53. Nat Rev Cancer.

[B4] Brooks CL, Gu W (2003). Ubiquitination, phosphorylation and acetylation: the molecular basis for p53 regulation. Curr Opin Cell Biol.

[B5] Lavin MF, Gueven N (2006). The complexity of p53 stabilization and activation. Cell Death Differ.

[B6] Marine J-C, Francoz S, Maetens M, Wahl G, Toledo F, Lozano G (2006). Keeping p53 in check: essential and synergistic functions of Mdm2 and MdmX. Cell Death Differ.

[B7] Toledo F, Wahl GM (2006). Regulating the p53 pathway: *in vitro *hypothesis, *in vivo veritas*. Nat Rev Cancer.

[B8] Marine J-C, Dyer MA, Jochemsen AG (2007). MDMX: from bench to bedside. J Cell Sci.

[B9] Wang YV, Wade M, Wong E, Li YC, Rodewald LW, Wahl GM (2007). Quantitative analyses reveal the importance of regulated Hdmx degradation for P53 activation. Proc Natl Acad Sci USA.

[B10] Xiong S, Van Pelt CS, Elizondo-Fraire AC, Liu G, Lozano G (2006). Synergistic roles of Mdm2 and Mdm4 for p53 inhibition in central nervous system development. Proc Natl Acad Sci USA.

[B11] Chen L, Gilkes DM, Pan Y, Lane WS, Chen J (2005). ATM- and Chk2-dependent phosphorylation of MDMX contribute to p53 activation after DNA damage. EMBO J.

[B12] Okamoto K, Kashima K, Pereg Y, Ishida M, Yamazaki S, Nota A, Teunisse A, Migliorini D, Kitabayashi I, Marine JC, Prives C, Shiloh Y, Jochemsen AG, Taya Y (2005). DNA damage-induced phosphorylation of MdmX at serine 367 activates p53 by targeting MdmX for Mdm2-dependent degradation. Mol Cell Biol.

[B13] Pereg Y, Shkedy D, de Graaf P, Meulmeester E, Edelson-Averbukh M, Salek M, Biton S, Teunisse AF, Lehmann WD, Jochemsen AG, Shiloh Y (2005). Phosphorylation of Hdmx mediates its Hdm2- and ATM-dependent degradation in response to DNA damage. Proc Natl Acad Sci USA.

[B14] Pereg Y, Lam S, Teunisse A, Biton S, Meulmeester E, Mittelman L, Buscemi G, Okamoto K, Taya Y, Shiloh Y, Jochemsen AG (2006). Differential roles of ATM- and Chk2-mediated phosphorylations of Hdmx in response to DNA damage. Mol Cell Biol.

[B15] Danovi D, Meulmeester E, Pasini D, Migliorini D, Capra M, Frenk R, de Graaf P, Francoz S, Gasparini P, Gobbi A, Helin K, Pelicci PG, Jochemsen AG, Marine JC (2004). Amplification of Mdmx (or Mdm4) directly contributes to tumor formation by inhibiting p53 tumor suppressor activity. Mol Cell Biol.

[B16] Backe J, Hofferbert S, Skawran B, Dörk T, Stuhrmann M, Karstens JH, Untch M, Meindl A, Burgemeister R, Chang-Claude J, Weber BHF (1999). Frequency of BRCA1 mutation 5382insC in German breast cancer patients. Gynecol Oncol.

[B17] Dörk T, Bendix R, Bremer M, Rades D, Klöpper K, Nicke M, Skawran B, Hector A, Yamini P, Steinmann D, Weise S, Stuhrmann M, Karstens JH (2001). Spectrum of ATM gene mutations in a hospital-based series of unselected breast cancer patients. Cancer Res.

[B18] The CHEK2 Breast Cancer Case-Control Consortium (2004). CHEK2*1100delC and susceptibility to breast cancer: a collaborative analysis involving 10,860 breast cancer cases and 9,065 controls from 10 studies. Am J Hum Genet.

[B19] Bogdanova N, Enßen-Dubrowinskaja N, Festchenko S, Lazijuk S, Rogov YI, Dammann O, Bremer M, Karstens JH, Sohn C, Dörk T (2005). Association of two mutations in the CHEK2 gene with breast cancer. Int J Cancer.

[B20] Bogdanova N, Feshchenko S, Cybulski C, Dörk T *CHEK2 *mutation and hereditary breast cancer. Journal of Clinical Oncology.

[B21] The Breast Cancer Association Consortium (2006). Commonly studied single-nucleotide polymorphisms and breast cancer: results from the Breast Cancer Association Consortium. J Natl Cancer Inst.

[B22] Cox A, Dunning AM, Garcia-Closas M, Balasubramanian S, Reed MWR, Pooley KA, Scollen S, Ponder BAJ, Chanock S, Lissowska J, Brinton L, Southey MC, Hopper JL, McCredie MRE, Giles GG, Fletcher O, Johnson N, dos Santos Silva I, Gibson L, Bojesen SE, Nordestgaard BG, Axelsson CK, Torres D, Hamann U, Justenhoven C, Brauch H, Chang-Claude J, Kropp S, Risch A, Wang-Gohrke S, Schürmann P, Bogdanova N, Dörk T, Fagerholm R, Aaltonen K, Blomqvist C, Nevanlinna H, Seal S, Stratton MR, Rahman N, Sangrajrang S, Hughes D, Odefrey F, Brennan P, Spurdle AB, Chenevix-Trench G, Beesley J, Mannermaa A, Hartikainen J, Kataja V, Kosma V-M, Couch FJ, Olson J, Goode EL, Broeks A, Schmidt MK, Hogervorst FBL, Van't Veer LJ, Kang D, Yoo K-Y, Noh D-Y, Ahn S-H, Wedrén S, Hall P, Low Y-L, Liu J, Milne RL, Ribas G, Gonzalez-Neira A, Benitez J, Sigurdson AJ, Stredrick DL, Alexander BH, Struewing JP, Pharoah PDP, Easton DF, The Breast Cancer Susceptibility Collaboration (UK), The Katherine Cunningham Foundation Consortium for Research into Familial Breast Cancer (2007). A common coding variant in CASP8 is associated with breast cancer risk. Nat Genet.

[B23] Easton DF, Pooley KA, Dunning AM, Pharoah PDP, Thompson D, Ballinger DG, Struewing JP, Morrison J, Field H, Luben R, Wareham N, Ahmed S, Healey CS, Bowman R, Meyer KB, Haiman CA, Kolonel LK, Henderson BE, Le Marchand L, Brennan P, Sangrajrang S, Gaborieau V, Odefrey F, Shen C-Y, Wu P-E, Wang H-C, Eccles D, Gareth Evans D, Peto J, Fletcher O, Johnson N, Seal S, Stratton MR, Rahman N, Chenevix-Trench G, Bojesen SE, Nordestgaard BG, Axelsson CK, Garcia-Closas M, Brinton L, Chanock S, Lissowska J, Peplonska B, Nevanlinna H, Fagerholm R, Eerola H, Kang D, Yoo K-Y, Noh D-Y, Ahn S-H, Hunter DJ, Hankinson SE, Cox DG, Hall P, Wedren S, Liu J, Low Y-L, Bogdanova N, Schürmann P, Dörk T, Tollenaar RAEM, Jacobi CE, Devilee P, Klijn JGM, Sigurdson AJ, Doody MM, Alexander BH, Zhang J, Cox A, Brock IW, MacPherson G, Reed MWR, Couch F, Goode EL, Olson JE, Meijers-Heijboer H, van den Ouweland A, Uitterlinden A, Rivadeneira F, Milne RL, Ribas G, Gonzalez-Neira A, Benitez J, Hopper JL, McCredie M, Southey M, Giles GG, Schroen C, Justenhoven C, Brauch H, Hamann U, Ko Y-D, Spurdle AB, Beesley J, Chen X, Mannermaa A, Kosma V-M, Kataja V, Hartikainen J, Day NE, Cox DR, Ponder BAJ, The S. E. A. R. C. H. collaborators, kConFab, A. O. C. S. Management Group (2007). A genome-wide association study identifies breast cancer susceptibility loci. Nature.

[B24] Yeo G, Burge CB (2004). Maximum entropy modeling of short sequence motifs with applications to RNA splicing signals. J Comput Biol.

[B25] Smith PJ, Zhang C, Wang J, Chew SL, Zhang MQ, Krainer AR (2006). An increased specificity score matrix for the prediction of SF2/ASF-specific exonic splicing enhancers. Hum Mol Genet.

[B26] Giglio S, Mancini F, Gentiletti F, Sparaco G, Felicioni L, Barassi F, Martella C, Prodosmo A, Iacovelli S, Buttitta F, Farsetti A, Soddu S, Marchetti A, Sacchi A, Pontecorvi A, Moretti F (2005). Identification of an Aberrantly Spliced Form of HDMX in Human Tumors: A New Mechanism for HDM2 Stabilization. Cancer Res.

[B27] Rallapalli R, Strachan G, Cho B, Mercer WE, Hall DJ (1999). A Novel MDMX Transcript Expressed in a Variety of Transformed Cell Lines Encodes a Truncated Protein with Potent p53 Repressive Activity. J Biol Chem.

[B28] Levy-Lahad E, Lahad A, Eisenberg S, Dagan E, Paperna T, Kasinetz L, Catane R, Kaufman B, Beller U, Renbaum P, Gershoni-Baruch R (2001). A single nucleotide polymorphism in the RAD51 gene modifies cancer risk in BRCA2 but not BRCA1 carriers. Proc Natl Acad Sci USA.

[B29] Wang WW, Spurdle AB, Kolachana P, Bove B, Modan B, Ebbers SM, Suthers G, Tucker MA, Kaufman DJ, Doody MM, Tarone RE, Daly M, Levavi H, Pierce H, Chetrit A, Yechezkel GH, Chenevix-Trench G, Offit K, Godwin AK, Struewing JP (2001). A single nucleotide polymorphism in the 5' untranslated region of RAD51 and risk of cancer among BRCA1/2 mutation carriers. Cancer Epidemiol Biomarkers Prev.

[B30] Kadouri L, Kote-Jarai Z, Hubert A, Durocher F, Abeliovich D, Glaser B, Hamburger T, Eeles RA, Peretz T (2004). A single-nucleotide polymorphism in the RAD51 gene modifies breast cancer risk in BRCA2 carriers, but not in BRCA1 carriers or noncarriers. Br J Cancer.

[B31] Jakubowska A, Gronwald J, Menkiszak J, Gorski B, Huzarski T, Byrski T, Edler L, Lubinski J, Scott RJ, Hamann U (2007). The RAD51 135G>C polymorphism modifies breast cancer and ovarian cancer risk in Polish BRCA1 mutation carriers. Cancer Epidemiol Biomarkers Prev.

[B32] Ng PC, Henikoff S (2002). Accounting for human polymorphisms predicted to affect protein function. Genome Res.

[B33] Bairoch A, Bucher P, Hofmann K (1997). The PROSITE database, its status in 1997. Nucl Acids Res.

[B34] Boersma B, Howe T, Goodman J, Yfantis HG, Lee DH, Chanock SJ, Ambs S (2006). Association of Breast Cancer Outcome With Status of p53 and MDM2 SNP309. J Natl Cancer Inst.

[B35] Bond GL, Hu W, Levine A (2005). A single nucleotide polymorphism in the MDM2 gene: from a molecular and cellular explanation to clinical effect. Cancer Res.

[B36] Bougeard G, Baert-Desurmont S, Tournier I, Vasseur S, Martin C, Brugieres L, Chompret A, Bressac-de Paillerets B, Stoppa-Lyonnet D, Bonaiti-Pellie C, Frebourg T (2006). Impact of the MDM2 SNP309 and p53 Arg72Pro polymorphism on age of tumour onset in Li-Fraumeni syndrome. J Med Genet.

[B37] Copson ER, White HE, Blaydes JP, Robinson DO, Johnson PW, Eccles DM (2006). Influence of the MDM2 single nucleotide polymorphism SNP309 on tumour development in BRCA1 mutation carriers. BMC Cancer.

[B38] Ohayon T, Gershoni-Baruch R, Papa MZ, Distelman Menachem T, Eisenberg Barzilai S, Friedman E (2005). The R72P P53 mutation is associated with familial breast cancer in Jewish women. Br J Cancer.

[B39] Osorio A, Martinez-Delgado B, Pollan M, Cuadros M, Urioste M, Torrenteras C, Melchor L, Diez O, De La Hoya M, Velasco E, Gonzalez-Sarmiento R, Caldes T, Alonso C, Benitez J (2006). A haplotype containing the p53 polymorphisms Ins16bp and Arg72Pro modifies cancer risk in BRCA2 mutation carriers. Hum Mutat.

[B40] NCBI SNP Database, as of May 25, 2006. http://www.ncbi.nlm.nih.gov/sites/entrez.

[B41] Chamary J-V, Parmley JL, Hurst LD (2006). Hearing silence: non-neutral evolution at synonymous sites in mammals. Nat Rev Genet.

[B42] Kotlar D, Lavner Y (2006). The action of selection on codon bias in the human genome is related to frequency, complexity, and chronology of amino acids. BMC Genomics.

[B43] Kimchi-Sarfaty C, Oh JM, Kim IW, Sauna ZE, Calcagno AM, Ambudkar SV, Gottesman MM (2007). A "silent" polymorphism in the MDR1 gene changes substrate specificity. Science.

[B44] Komar AA (2007). SNPs, silent but not invisible. Science.

[B45] TFSearch v1.3. http://www.cbrc.jp/htbin/nph-tfsearch.

[B46] Ahmed K, Gerber DA, Cochet C (2002). Joining the cell survival squad: an emerging role for protein kinase CK2. Trends Cell Biol.

[B47] Meggio F, Pinna LA (2003). One-thousand and one substrates of protein kinase CK2?. FASEB J.

[B48] Shanware NP, Trinh AT, Williams LM, Tibbetts RS (2007). Coregulated ataxia telangiectasia-mutated and casein kinase sites modulate cAMP-response element-binding protein-coactivator interactions in response to DNA damage. J Biol Chem.

[B49] Chen L, Li C, Pan Y, Chen J (2005). Regulation of p53-MDMX interaction by casein kinase I alpha. Mol Cell Biol.

[B50] Human Genome Variation Society. http://www.hgvs.org/mutnomen/.

